# Effects of Fermentation and Enzymatic Hydrolysis of Cottonseed Protein on Rumen Fermentation Characteristics, Intestinal Barrier Function, and Hepatic Metabolism in Suckling Lambs

**DOI:** 10.3390/ani15182652

**Published:** 2025-09-10

**Authors:** Weidong Niu, Changzhao Jin, Xiaohan Fan, Haiyun Yang, Yong Chen, Jiancheng Liu

**Affiliations:** Research Center for Biological Feed and Animal Gut Health, College of Animal Science, Xinjiang Agricultural University, Urumqi 830052, China; nwd163com@163.com (W.N.); j17799683492@163.com (C.J.);

**Keywords:** fermentation, enzymatic hydrolysis, cottonseed protein, suckling lambs, rumen microbiota, hepatic metabolism

## Abstract

The research aimed to examine the effects of fermentation and enzymatic hydrolysis of cottonseed protein on body weight changes, serum biochemistry, rumen function, intestinal health, and liver metabolism of suckling lambs. A total of twelve 7-day-old healthy male Hu sheep body weights (5.27 ± 0.48 kg) were randomly distributed into two groups. Starter feed regimens containing microbial fermentation of cottonseed protein (MFCP) or enzymatic hydrolysate of cottonseed protein (EHCP) were administered to lambs during the initial 60-day period. Results showed that compared with EHCP group, the serum glucose, ruminal acetic, propionic, butyric and valeric acids concentrations, jejunal immunoglobulin G content and mRNA expressions of *Claudin 1* and *Occludin*, as well as the relative abundance of *actinobacteriota* and *pseudoscardovia* in the rumen were significantly increased in the MFCP group (*p* < 0.05), whereas an opposite trend was observed in the jejunum. *α*-amylase and trypsin enzymatic activities were observed between the two groups. Relative to EHCP treatment, the MFCP group exhibited 69 elevated and 103 reduced hepatic metabolites, and these metabolites displayed distinct enrichment patterns within specific metabolic networks, including *fructose and mannose metabolism* (*p* = 0.003), *arachidonic acid metabolism* (*p* = 0.017), *glycerophospholipid metabolism* (*p* = 0.036), and the *cAMP signaling pathway* (*p* = 0.047). Overall, microbial fermentation of cottonseed protein may be beneficial for strengthening intestinal barrier function and facilitating hepatic lipid metabolism and immune regulation, while enzymatic hydrolysis of cottonseed protein enhances gastrointestinal digestive enzyme activity, thereby promoting nutrient digestion of suckling lambs.

## Simple Summary

The rising cost of soybean meal has compelled the search for alternatives such as cottonseed protein, yet its application is constrained by the presence of large-molecule proteins and carbohydrates that are difficult to digest, alongside anti-nutritional factors. This study compared the effects of microbial fermentation treatment (MFCP) versus enzymatic hydrolysis treatment (EHCP) in newborn lamb feed. MFCP promoted rumen development, intestinal immunity, and hepatic fatty acid metabolism; EHCP improved gastrointestinal digestive function. Both methods effectively enhanced cottonseed protein’s suitability as animal feed.

## 1. Introduction

Currently, many countries primarily rely on soybean meal (SBM), derived from genetically modified soybeans, as the main plant protein source in animal feeds [1]. However, the soybean meal supply chain is highly susceptible to geopolitical disruptions, resulting in severe price volatility. The persistent increase in SBM prices presents substantial obstacles to the sustainable growth of livestock production [2]. Hence, there is a pressing necessity to discover viable substitutes that can successfully replace SBM to satisfy the plant-based protein requirements of livestock [3]. Cottonseed meal (CSM) represents the world’s second major plant-derived protein source for livestock feed following SBM [4], with global cottonseed output projected to attain 26.32 million metric tons. in the 2024–2025 period [5]. CSM exhibits elevated protein levels, a reasonably well-balanced amino acid composition, and widespread accessibility, positioning it as one of the most viable plant-based protein alternatives [6]. However, extensive deployment of cottonseed meal in animal feeds faces restrictions due to various impediments, notably the occurrence of deleterious substances like gossypol, cyclopropenoid fatty acids, phytic acid, and non-starch polysaccharides, combined with suboptimal protein absorption and compromised carbohydrate utilization [7]. Extensive research has revealed that thermal [8], solvent-based [9,10], and microbial treatment approaches [11,12] can successfully diminish antinutritional factors in CSM and improve its nutritional value, thus facilitating its application in livestock feeding programs. However, compared with conventional physical and chemical methods, biological treatments (fermentation and enzymatic hydrolysis) have become a major focus of current research on improving the utilization of cottonseed meal [13], owing to their environmental friendliness and high efficiency. The fermentative process harnesses favorable microorganisms that synthesize multiple enzyme types throughout their developmental phase, consequently decreasing antinutritional substance concentrations in CSM. Simultaneously, it increases the abundance of probiotics and generates bioactive nutrients such as small peptides, amino acids, organic acids, vitamins, and unidentified growth factors, ultimately enhancing the nutritional value of CSM [14]. According to existing literature, solid-state fermentation of CSM using different microorganisms—such as *Saccharomyces cerevisiae*, *Enterococcus faecalis*, and *Lactobacillus plantarum*—can significantly enhance the quality of CSM [15]. Research indicates that fermented cottonseed meal demonstrates favourable effects when applied to poultry, ruminants, and aquatic animals. Microbial fermentation of cottonseed meal enhances growth performance, ruminal volatile fatty acids, improves nutrient absorption and gut health, and reduces inflammation and physical barrier damage [16,17,18]. Moreover, the fermentation process is complex, time-consuming, and energy-intensive [6]. Enzymatic hydrolysis involves the degradation of cottonseed meal using enzymes such as proteases, cellulases, non-starch polysaccharidases, laccases, and compound enzymes under optimal conditions. On one hand, this approach degrades anti-nutritional factors [11]; Simultaneously, it fragments high-molecular-weight substances into lower molecular components that exhibit improved bioavailability and uptake by the host organism. Furthermore, enzymatic hydrolysis can compensate for the insufficiency of endogenous enzymes in animals or reduce their endogenous enzyme production, thereby allowing more energy and amino acids to be utilized for growth [19]. Compared to fermentation, enzymatic hydrolysis is more specific, more efficient, operates under milder reaction conditions, and better preserves the nutritional integrity of feed ingredients. Investigations into the incorporation of enzymatically processed CSM in avian species, swine, and aquatic animals have shown that it can enhance digestive enzyme secretion in young animals, alleviate digestive burden, increase feed intake and feed efficiency [20], promote intestinal structural development [21], improve antioxidant capacity [22], strengthen immune responses [23], and modulate the equilibrium of gut microbiome [24]. Furthermore, scholarly work has confirmed that both biofermentation and enzymatic cleavage of cottonseed protein possess the ability to form an array of physiologically active peptides, comprising free radical-neutralizing peptides [25], bactericidal peptides [26], immunomodulatory peptides [27], and antihypertensive peptides [28]. These bioactive peptides can effectively modulate physiological functions and promote overall health in animals.

As young ruminants, suckling lambs have underdeveloped rumens and intestines during early weaning, coupled with low immunity, making them susceptible to disease. Introducing solid feed during the early life stage of ruminants can improve rumen microbiota and tissue development [29]. However, the application of enzymatically hydrolyzed or fermented cottonseed protein products in starter diets for suckling lambs has been rarely reported. Therefore, this study aims to address the challenges posed by the presence of anti-nutritional factors in cottonseed protein and the low digestibility of its protein and carbohydrate components. The nutritional enhancement of cottonseed protein through microbial fermentation and complex enzymatic treatment was assessed. Additionally, the modified cottonseed protein was integrated into the early-stage diet of milk-fed lambs to evaluate its influence on growth indicators, serum biochemical markers, rumen fermentation parameters, gastrointestinal digestive enzymes, liver metabolism, jejunal mucosal immunoglobulin, and tight junction proteins. The final objective is to establish a scientific basis for utilizing biotreated cottonseed protein in enhancing digestive tract development and optimal growth in juvenile ruminants. The effects of fermented versus enzymatically hydrolysed cottonseed protein on suckling lambs may be as follows: fermented cottonseed protein may promote rumen development more favourably than its hydrolysed counterpart due to earlier microbial colonisation, whilst the latter may enhance digestive enzyme activity through the introduction of exogenous enzymes.

## 2. Materials and Methods

### 2.1. Ethical Statement

All animal experiments were conducted according to the relevant national guidelines and were approved by the Animal Welfare Care and Use Committee of Xinjiang Agricultural University (Xinjiang, China) (Animal protocol number: 2024006). Sampling operations were strictly conducted in accordance with the relevant provisions of the “Guiding Opinions on the Humane Treatment of Experimental Animals” issued by the Ministry of Science and Technology of the People’s Republic of China (Guo Ke Fa Cai Zi [2006]. No. 398).

### 2.2. Experimental Materials

Cottonseed protein utilized in this investigation (crude protein content: 59.60%; free gossypol [FG] content: 412.57 mg/kg) was purchased from Xinjiang Tycoon Group Co., Ltd., (Changji, China). The alkaline protease was sourced from Suntaq Bioscience Co., Ltd., (Guangzhou) and laccase was procured from Sunson Enzymes Co., Ltd., (Cangzhou, China). The strains of *Saccharomyces cerevisiae*, *Lactobacillus acidophilus*, and *Lactiplantibacillus plantarum* were provided by the laboratory of Xinjiang Agricultural University.

The preparation process for the fermented and enzymolyzed protein is as follows:

Exactly 100 g of cottonseed protein was measured and completely blended with 60 mL of sterilized distilled water. The blended solution was moved to a 500 mL Erlenmeyer flask. The pH value of cottonseed meal dissolved in water is between 6.0 and 6.3, which is not adjusted during the fermentation and enzymatic hydrolysis processes. For microbial fermentation, inocula of *Saccharomyces cerevisiae* (3.0 × 10^9^ CFU/mL), *Lactobacillus acidophilus* (1.0 × 10^9^ CFU/mL), and *Lactiplantibacillus plantarum* (3.0 × 10^8^ CFU/mL) cultured to the late logarithmic growth phase were added at 1% (*v*/*w*) each. For enzymatic hydrolysis, alkaline protease and laccase were added separately at 1% (*w*/*w*) each. After uniform blending, the flasks were maintained in a thermostat incubator at 37 °C, with shaking once every 24 h. The fermentation or enzymatic hydrolysis was conducted for a total duration of 72 h. Following treatment, the samples were dried in an oven at 60 °C for 24 h, ground, and passed through an 80-mesh sieve prior to nutritional analysis. The amino acid content (Table S1) and nutritional composition of cottonseed protein after fermentation or enzymatic hydrolysis are provided in Table 1.

### 2.3. Experimental Design and Feeding Management

Two experimental groups were established using twelve 7-day-old male Hu lambs (starting body weight: 5.27 ± 0.48 kg) through random allocation, with each group comprising six replicates containing one lamb each. The lambs were fed starter diets formulated with either MFCP or EHCP as the sole nitrogen source from day 7 to day 60 of age. Starter diets were prepared in compliance with the Chinese national standard *concentrated feed of sheep* (GB/T 20807-2006) [30], and Table 2 displays their ingredient composition and nutrient specifications. From day 1 to day 7, the lambs suckled from their dams. Beginning on day 7, lambs received artificial feeding with equivalent milk replacer volumes delivered thrice daily at morning (08:00), afternoon (14:00), and evening (20:00), while unrestricted availability of starter feed and drinking water was maintained throughout other periods. Lamb diarrhea was observed and recorded daily between 10:00 and 12:00 and 16:00 and 18:00, and faecal scoring (0 = formed, 1 = sticky or moderately formed, 2 = loose or mildly diarrheic, 3 = watery) was conducted. Faecal scores ≥ 2 defined diarrhea.

### 2.4. Sample Collection and Index Determination

#### 2.4.1. Body Weight Changes

On the seventh day after birth, lambs were individually weighed prior to morning feeding to establish their initial body weight. At sixty days of age, lambs were fasted for twelve hours before being weighed to determine their final body weight. Daily observation of diarrhoea occurrence in each lamb shall be conducted, with diarrhoea determined according to established criteria. The incidence rate shall be calculated based on the number of days each lamb exhibits diarrhoea.

$$\mathrm{Average}\;\mathrm{Daily}\;\mathrm{Gain}\;(\mathrm{ADG})=\frac{Final\;Body\;Weight\left(g\right)-Initial\;Body\;Weight\left(g\right)}{Number\;of\;Experimental\;Days}$$

$$\mathrm{Diarrhea}\;\mathrm{Rate}\;\left(\%\right)=\frac{\mathrm{Number}\;\mathrm{of}\;\mathrm{Days}\;\mathrm{With}\;\mathrm{Diarrhea}}{\mathrm{Number}\;\mathrm{of}\;\mathrm{Experimental}\;\mathrm{Days}}\;\times \;100$$

#### 2.4.2. Serum Biochemical Parameters

On day 60, the lambs underwent a 12 h fast prior to weighing, while blood collection was performed via jugular venipuncture to obtain serum. Serum levels of total protein (TP), albumin (ALB), globulin (GLB), blood urea nitrogen (BUN), glucose (GLU), total bilirubin, triglycerides (TG), total cholesterol (T-CHO), high-density lipoprotein cholesterol (HDL-c), low-density lipoprotein cholesterol (LDL-c), alanine aminotransferase (ALT), and aspartate aminotransferase (AST) were measured using a fully automated biochemical analyzer (TBA-FX8, Medical Imaging System, Canon Medical Systems Corporation, Ota, Tokyo, Japan).

#### 2.4.3. Rumen Fermentation Parameters

After fasting and body weight measurement at 60 days of age, all lambs were euthanized by exsanguination via the jugular vein. Upon rapid opening of the abdominal cavity, dual 50 mL portions of ruminal contents were sampled. These specimens were cryopreserved in liquid nitrogen and kept at −80 °C. External standard gas chromatography (GC-2010, Shimadzu, Kyoto, Japan) was utilized for volatile fatty acids (VFAs) quantification in ruminal contents, allowing calculation of total volatile fatty acids (TVFAs) concentrations and the ratio of acetate to propionate [31]. The phenol-sodium hypochlorite colorimetric technique was applied for NH_3_-N measurement [32]. Lactic acid content was analyzed via a lactate analyzer (LM5, Analox, Hammersmith, London, UK).

#### 2.4.4. Rumen Microbial Diversity

Rumen microbial sequencing and diversity analysis were conducted by Novogene Co., Ltd. (Beijing, China). The V3–V4 hypervariable regions of the 16S rRNA gene were amplified using the primers 341F (5′-CCTAYGGGRBGCASCAG-3′) and 806R (5′-GGACTACNNGGGTATCTAAT-3′) for paired-end sequencing [33]. The 30 µL PCR reaction system contained 15 µL of Phusion^®^ High-Fidelity PCR Master Mix (New England Biolabs, Beijing, China), 0.2 µM of each primer, and 10 ng of genomic DNA. Raw sequencing reads were assembled using FLASH (v1.2.11) (http://ccb.jhu.edu/software, accessed on 10 November 2024) and subjected to quality filtering using Fastp (v0.23.1) to obtain high-quality sequences [34]. Chimeric sequences were identified and removed using the SILVA database (v138), resulting in optimized sequences for downstream analysis. All samples were uniformly pooled to yield 18,076 valid sequences prior to subsequent analysis. Amplicon sequence variants (ASVs) were generated using the DADA2 algorithm implemented in QIIME2 (v1.9.1) [35]. Taxonomic annotation of microbial sequences was performed using the built-in classifier in QIIME2.

#### 2.4.5. Digestive Enzyme Activities in the Abomasum, Duodenum, and Jejunum Contents

After slaughter, contents from the abomasum, mid-duodenum, and mid-jejunum were harvested, flash-frozen using liquid nitrogen, and preserved at −80 °C. Intestinal content analysis for α-amylase (C016-1, colorimetric method), lipase (A054-2, methyl resorufin substrate method), trypsin (A080-2, UV colorimetric method) activities, and total protein concentration (A045-4, BCA microplate method) was performed using commercial assay systems from Nanjing Jiancheng Bioengineering Institute (Nanjing, China).

#### 2.4.6. Expression of Immunoglobulins and Tight Junction Protein mRNA in Jejunal Mucosa

After slaughter, mucosal samples were scraped from the mid-jejunum using sterile microscope slides, snap-frozen in liquid nitrogen, and stored at −80 °C. The concentrations of secretory immunoglobulin A (SIgA), immunoglobulin G (IgG), and immunoglobulin M (IgM) in the mucosa were determined using ELISA kits supplied by Shanghai MLBio Co., Ltd. (Shanghai, China). The recommended concentration ranges for lamb SIgA, IgG, and IgM are 1.5–48 μg/mL, 2.5–80 mg/mL, and 0.075–2.4 mg/mL, respectively. The mRNA transcript levels of tight junction-associated proteins zonula occludens-1 (ZO-1), claudin-1, and occludin were determined through services provided by Beijing Sino-UK Institute of Biological Technology (Beijing, China). Briefly, the target gene sequences were obtained from the GenBank database, and specific primers were designed using Primers Express 6.0 software. Primer sequences are listed in Table 3. Total RNA isolation from jejunal mucosal tissues was accomplished with TRIzol reagent kit, and subsequent complementary DNA (cDNA) generation was carried out via reverse transcription following the RevertAid First Strand cDNA Synthesis Kit manufacturer’s guidelines. cDNA served as the template for quantitative real-time PCR (qRT-PCR) analysis, with *β*-actin used as the housekeeping gene. The amplification process utilized FastStart Universal SYBR Green Master Mix as per manufacturer guidelines. Relative quantification of target gene mRNA expression was achieved through the 2^−ΔΔCt^ analytical approach.

#### 2.4.7. Hepatic Untargeted Metabolomics

After slaughter, approximately 10 g of liver tissue was collected from each experimental lamb. Surface moisture was blotted with sterile filter paper, with tissue samples immediately cryopreserved in liquid nitrogen and maintained at −80 °C within sterile cryovials. The cryopreserved hepatic tissue was mechanically disrupted using a mortar and pestle apparatus precooled by liquid nitrogen, resulting in homogeneous powder. The powdered specimen (100 mg) was thoroughly mixed via vortexing with 500 μL of chilled 80% methanol containing 0.1% formic acid. Following a 5 min incubation period on ice, the mixture underwent centrifugation at 15,000× *g* for 20 min under 4 °C conditions. The supernatant fraction was subjected to dilution with LC-MS grade water until the methanol concentration reached 53%, then processed through a secondary centrifugation. Filtration of the prepared solution was conducted using a 0.22 μm membrane, with the filtrate employed as the injection sample for LC-MS determination. Quality control (QC) samples were generated by pooling all individual specimens in parallel, serving to evaluate system stability and data reproducibility across the entire analytical procedure. Metabolomic profiling of liver samples was implemented through a Thermo Vanquish UHPLC system integrated with a Q Exactive HF mass spectrometer (Thermo Fisher Scientific, Bremen, Germany). The separation was facilitated by a hypersil GOLD C18 column (100 × 2.1 mm, 1.9 μm) operating at 40 °C. The mobile phases consisted of 0.1% formic acid in water (solvent A) and methanol (solvent B). The gradient elution program was as follows: 0–1.5 min, 2% B; 1.5–3 min, linear ramp to 85% B; 3–10 min, increased to 100% B and maintained until 12 min, followed by re-equilibration. The instrument was configured in electrospray ionization (ESI) mode with *m*/*z* detection spanning 100–1500. ESI source parameters were established as: spray voltage 3.5 kV, sheath gas flow 35 psi, auxiliary gas flow 10 L/min, ion transfer tube temperature 320 °C, and auxiliary heater temperature 350 °C. Tandem mass spectrometry (MS/MS) data collection employed a data-dependent acquisition (DDA) approach, utilizing collision energy that was adjusted through a stepwise gradient methodology [36].

### 2.5. Statistical Analysis

Experimental data were organized using Microsoft Excel and statistically analyzed with SPSS version 29.0 (IBM Corp., Armonk, NY, USA). Independent-sample *t*-tests were employed to evaluate differences in body weight changes, serum biochemical parameters, rumen fermentation characteristics, gastrointestinal digestive enzyme activities, intestinal immune indicators, and tight junction gene expression levels, with multiple comparison corrections applied (*p* < 0.05). The analytical framework designated *p* < 0.05 as the threshold for statistical importance, *p* < 0.01 for markedly substantial effects, and 0.05 ≤ *p* < 0.10 for indicating marginal statistical relevance. Rumen microbial data were processed using Qiime2 for calculating α-diversity indices. The evaluation of *β*-diversity was carried out using UniFrac distance calculations with and without phylogenetic weighting. The “ade4” and “ggplot2” packages in R software (version 4.0.3) were used for visualization and statistical comparison of microbial community differences via *t*-tests. Metabolomics data were normalized using the metaX software (version 4.0.3)package. Principal component analysis (PCA) and orthogonal partial least squares discriminant analysis (OPLS-DA) methodologies were employed to determine variable importance in projection (VIP) scores for variable importance assessment. Metabolite screening for differential expression employed combined thresholds including VIP > 1, statistical relevance at *p* < 0.05, and fold change (FC) ratios ≥ 2 or ≤0.5.

## 3. Results

### 3.1. Effects of Fermentation and Enzymatic Hydrolysis of Cottonseed Protein on Body Weight Changes in Suckling Lambs

Table 4 demonstrates that no meaningful disparities were detected between the MFCP and EHCP treatments concerning body weight parameters, ADG, and diarrhea incidence in suckling lamb populations.

### 3.2. Effects of Fermentation and Enzymatic Hydrolysis of Cottonseed Protein on Serum Biochemical Parameters in Suckling Lambs

Data shown in Table 5 revealed that the MFCP group exhibited substantially greater serum glucose concentrations relative to the EHCP group (*p* < 0.01). Nevertheless, no notable differences were observed between the two treatments regarding serum concentrations of total protein, albumin, globulin, blood urea nitrogen, total bilirubin, alanine aminotransferase (ALT), aspartate aminotransferase (AST), total cholesterol, triglycerides, high-density lipoprotein cholesterol (HDL-c), and low-density lipoprotein cholesterol (LDL-c).

### 3.3. Effects of Fermentation and Enzymatic Hydrolysis of Cottonseed Protein on Rumen Fermentation Parameters in Suckling Lambs

Table 6 data revealed that acetic acid, propionic acid, butyric acid, valeric acid, and TVFA concentrations in rumen contents were markedly elevated in the MFCP group compared to the EHCP group (*p* < 0.01). While the acetate-to-propionate ratio was greater in the MFCP treatment, this difference lacked statistical significance. Both groups exhibited comparable levels of isobutyric acid, isovaleric acid, NH_3_-N, and lactic acid in rumen contents.

### 3.4. Effects of Fermentation and Enzymatic Hydrolysis of Cottonseed Protein on Rumen Microbial Diversity in Suckling Lambs

**Figure 1:**
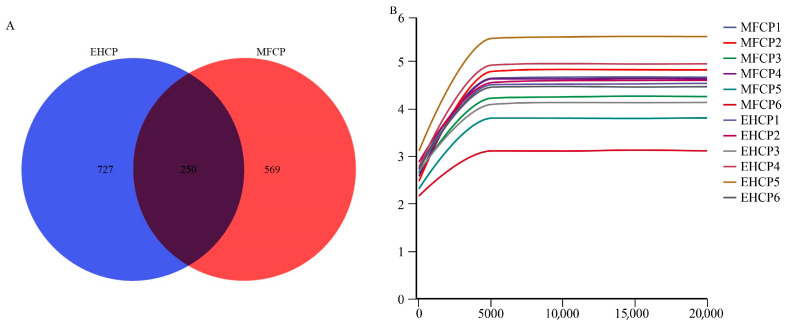
Venn diagram and dilution curve of rumen microbiota in suckling lambs. (**A**): Number of rumen microbiota ASVs, (**B**): Rumen microbiota dilution curve. MFCP: Microbial Fermentation of Cottonseed Protein; EHCP: Enzymatic Hydrolysate of Cottonseed Protein.

Analysis of rumen microbiota ASVs from both treatment groups identified a total of 1546 ASVs. Of these, 250 ASVs were common to both MFCP and EHCP treatments, representing 16.17% of all detected ASVs. The MFCP group had 569 unique ASVs (36.81% of the total), while the EHCP group exhibited 727 unique ASVs (47.03% of the total) (Figure 1A). Rarefaction curves leveled off at a sequencing depth of 18,076 reads, indicating that species richness and diversity had reached saturation and the sequencing coverage was sufficient across all samples (Figure 1B).

**Figure 2:**
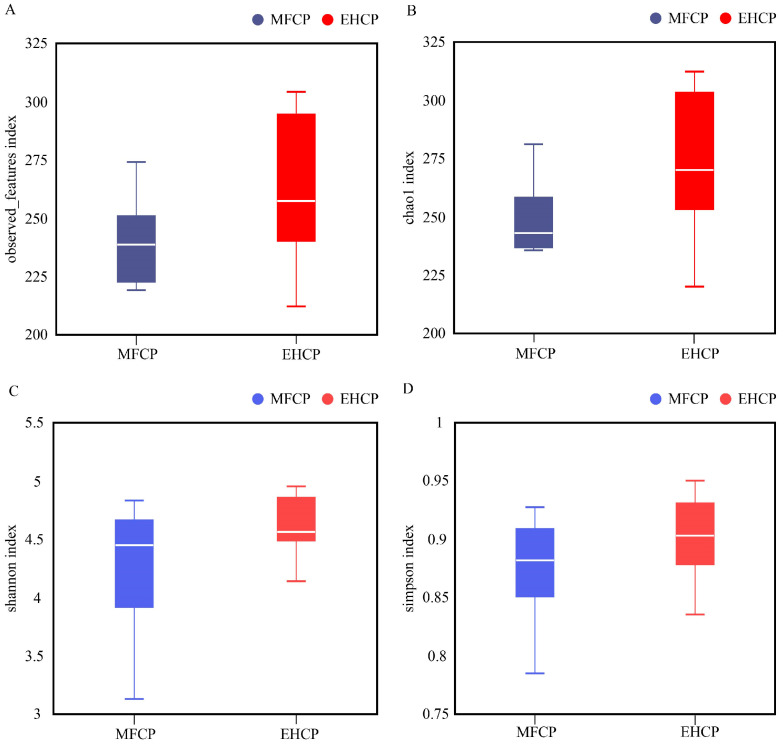
Effects of fermentation and enzymatic hydrolysis of cottonseed protein on the α diversity index of rumen microbiota in suckling lambs. (**A**): Observed features index; (**B**): Chao1 index; (**C**): Shannon index; (**D**): Simpson index. MFCP: Microbial Fermentation of Cottonseed Protein; EHCP: Enzymatic Hydrolysate of Cottonseed Protein.

**Figure 3:**
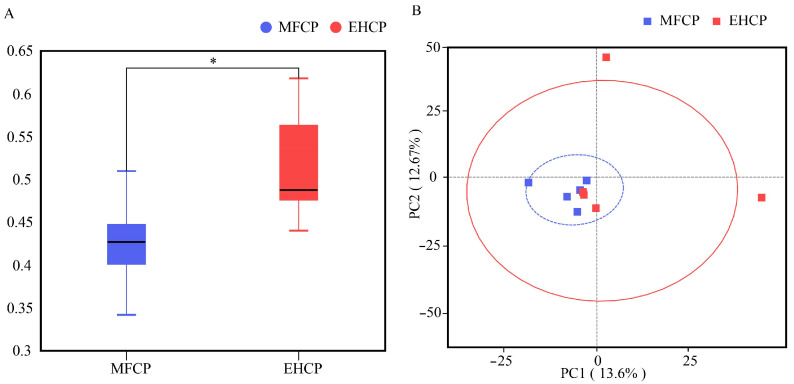
Effects of fermentation and enzymatic hydrolysis of cottonseed protein on the β-diversity index of rumen microbiota in suckling lambs. (**A**): Beta diversity index analysis chart; (**B**): PCA chart. MFCP: Microbial Fermentation of Cottonseed Protein; EHCP: Enzymatic Hydrolysate of Cottonseed Protein. * means *p* < 0.05.

According to Figure 2, both MFCP and EHCP groups exhibited comparable α-diversity indices, encompassing Observed_features, Chao1, Shannon, and Simpson measurements. Nevertheless, Figure 3A demonstrates that rumen microbiota β-diversity showed marked differences between the two treatments. PCoA analysis of the β-diversity matrix displayed distinct spatial separation of microbial community structures comparing MFCP and EHCP groups, with samples from the two treatments exhibiting distinct clustering on the PC1–PC2 plane (Figure 3B).

**Figure 4:**
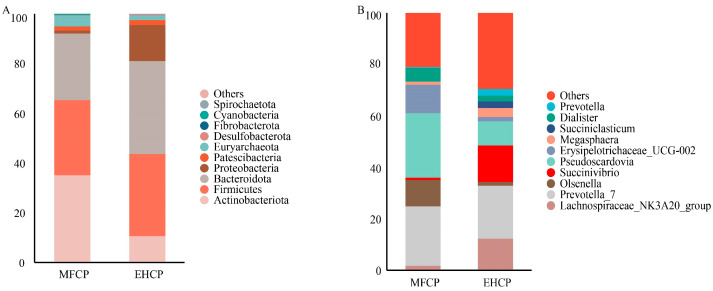
Effects of fermentation and enzymatic hydrolysis of cottonseed protein on the top 10 phyla of rumen microbiota in suckling lambs. (**A**): Top 10 rumen microorganisms by genus; (**B**): Top 10 rumen microorganisms by species. MFCP: Microbial Fermentation of Cottonseed Protein; EHCP: Enzymatic Hydrolysate of Cottonseed Protein.

Taxonomic annotation identified the top 10 microbial taxa at both the phylum and genus levels (Tables S2 and S3). At the phylum level (Figure 4A), *Actinobacteriota* was the most dominant phylum, showing a significantly higher relative abundance in the MFCP group compared to the EHCP group. In contrast, *spirochaetota* was notably more prevalent in EHCP group animals versus those in the MFCP group. Figure 4B illustrates that the relative abundances of *pseudoscardovia* and *erysipelotrichaceae_UCG-002* were substantially higher in MFCP-treated animals, while the EHCP group exhibited considerably increased *prevotella* levels.

**Figure 5:**
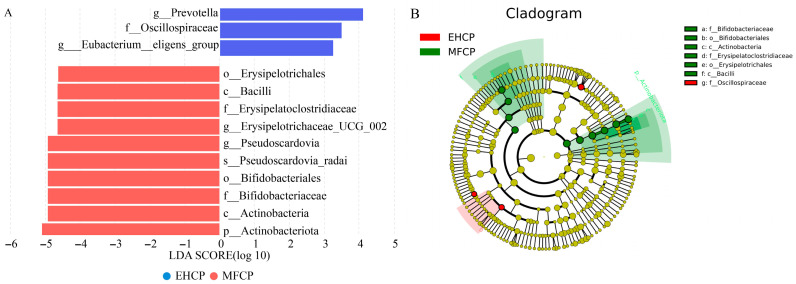
Histogram and cladogram of rumen microbiota distribution in suckling lambs after fermentation and enzymatic hydrolysis of cottonseed protein. (**A**): Histogram. (**B**): Cladogram. MFCP: Microbial Fermentation of Cottonseed Protein; EHCP: Enzymatic Hydrolysate of Cottonseed Protein.

Based on LEfSe analysis, a histogram (Figure 5A) and cladogram (Figure 5B) were produced utilizing Linear Discriminant Analysis (LDA) scores above 3.0 to determine bacterial taxa displaying notably distinct abundances between the two groups. Analysis revealed 13 bacterial taxa spanning various taxonomic ranks that exhibited differential abundance patterns between the MFCP and EHCP groups. Among these taxa, 10 showed greater representation in the MFCP group and three demonstrated higher abundance in the EHCP group. As illustrated in Figure 5A, the MFCP group exhibited substantial enrichment of actinobacteria and its subordinate taxonomic levels, including the class *actinobacteria*, the order *bifidobacteriales*, and the family *Bifidobacteriaceae* (LDA > 4). Additionally, *pseudoscardovia* and its species-level taxon *pseudoscardovia radai* were specifically enriched in the MFCP group (LDA > 4). Furthermore, *erysipelatoclostridiaceae* and *erysipelotrichales* were significantly enriched in the MFCP group (LDA > 4). *prevotella*, *oscillospiraceae,* and *eubacterium_eligens_group* also showed significant enrichment trends in the MFCP group (LDA > 3).

### 3.5. Effects of Fermentation and Enzymatic Hydrolysis of Cottonseed Protein on Gastrointestinal Digestive Enzyme Activities in Suckling Lambs

Table 7 reveals that *α*-amylase activity in abomasal contents, as well as *α*-amylase and trypsin activities in jejunal contents, were considerably more pronounced in the EHCP group than in the MFCP group. In contrast, both groups exhibited comparable activities of lipase and trypsin in the abomasum and *α*-amylase, lipase, and trypsin in the duodenal contents, with no substantial differences detected. While the EHCP group exhibited elevated lipase activity in jejunal contents compared to the MFCP group, this difference lacked statistical relevance.

### 3.6. Effects of Fermentation and Enzymatic Hydrolysis of Cottonseed Protein on Immunoglobulin Concentrations and Tight Junction Protein Gene Expression in Jejunal Mucosa of Suckling Lambs

**Figure 6:**
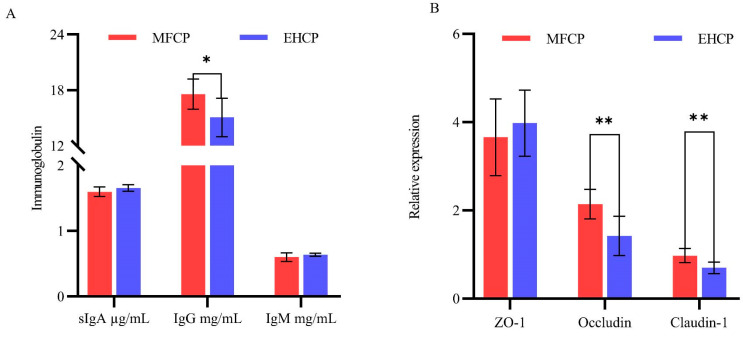
Effects of fermentation and enzymatic hydrolysis of cottonseed protein on immunoglobulin concentration and tight junction protein gene expression in the jejunal mucosa of lactating lambs. (**A**): Jejunum mucosa immunoglobulin; (**B**): Jejunum mucosa tight junction protein gene expression level. * Indicates *p* < 0.05, significant difference; ** indicates *p* < 0.01, extremely significant difference. MFCP: Microbial Fermentation of Cottonseed Protein; EHCP: Enzymatic Hydrolysate of Cottonseed Protein.

As shown in Figure 6A (Table S4), the concentration of IgG in the jejunal mucosa was significantly higher in the MFCP group compared to the EHCP group; conversely, the two groups exhibited statistically indistinguishable levels of sIgA and IgM. According to Figure 6B, the two groups exhibited statistically indistinguishable ZO-1 mRNA expression levels. Nevertheless, the MFCP group demonstrated markedly elevated expression levels of Occludin and Claudin-1 compared to the EHCP group (*p* < 0.01).

### 3.7. Effects of Fermentation and Enzymatic Hydrolysis of Cottonseed Protein on Hepatic Metabolism in Suckling Lambs

#### 3.7.1. Identification of Hepatic Metabolites and Analysis of Differential Metabolites

**Figure 7:**
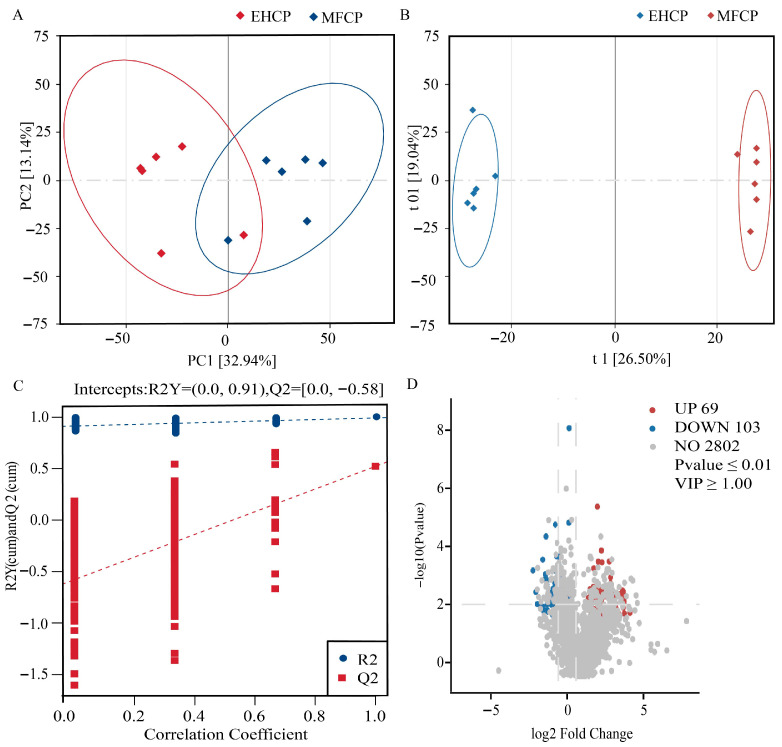
PCA scatter plot of the effects of fermentation and enzymatic hydrolysis of cottonseed protein on liver metabolism in lactating lambs, scatter plot obtained from the OPLS-DA model analysis, and volcano plot of differentially expressed metabolites. (**A**): PCA plot of the overall sample; (**B**): Scatter plot of the OPLS-DA model; (**C**): Permutation test plot of the MFCP group and EHCP group; (**D**): Volcano plot of differential metabolites with thresholds of FC > 1.5, VIP > 1, and *p* < 0.01. R2Y is the model’s explanatory power for the sample grouping matrix; Q2Y is the model’s predictive ability. MFCP: Microbial Fermentation of Cottonseed Protein; EHCP: Enzymatic Hydrolysate of Cottonseed Protein.

Figure 7A demonstrates how the PCA score plot of all liver samples showed each sample contained in the 95% confidence interval, thereby substantiating the experimental data integrity. The OPLS-DA score plots (Figure 7B,C) revealed distinct clustering patterns when comparing MFCP and EHCP groups. The results of permutation testing confirmed the validity, stability, and reliability of the model. Differential metabolites between groups were selectively identified based on LC-MS analysis using the following thresholds: fold change (FC) > 1.5, VIP > 1, and *p* < 0.01. The analysis presented in Figure 7D revealed 2974 metabolites in liver samples, where 172 exhibited differential regulation. Of these, 69 metabolites were significantly upregulated (Table S5), including *palmitoleic acid*, *conjugated linoleic acids* (CLA), *19s-hydroxyeicosatetraenoic acid* (19S-HETE), *arachidonoyl ethanolamide*, and *13(s)-hydroxyoctadecadienoic acid* (13(S)-HODE). Additionally, 103 metabolites were significantly downregulated (Table S6), including *d-fructose-6-phosphate*, *lysoPC (14:0*/*0:0*), *dihydrozeatin*, *mycophenolate mofetil*, and *d-fructose*.

#### 3.7.2. Functional Enrichment Analysis of Differential Metabolites

**Figure 8:**
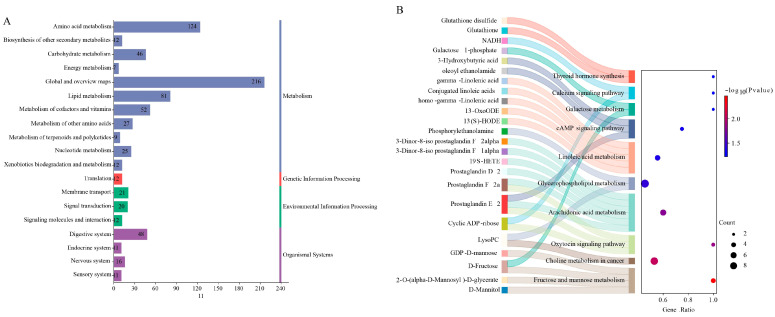
KEGG pathway annotation diagram and Sankey bubble diagram of fermentation and enzymatic hydrolysis of cottonseed protein on liver metabolism in suckling lambs. (**A**): KEGG pathway annotation diagram of the MFCP vs. EHCP group; (**B**): Sankey bubble diagram of the MFCP vs. EHCP group; MFCP: Microbial Fermentation of Cottonseed Protein; EHCP: Enzymatic Hydrolysate of Cottonseed Protein.

The analysis in Figure 8A shows twenty highly enriched metabolic pathways, with primary focus on *amino acid metabolism*, *global and overview maps*, *lipid metabolism*, *metabolism of cofactors and vitamins*, and *digestive system* processes. Differential metabolites underwent KEGG pathway enrichment analysis, as demonstrated in Figure 8B. Comparative analysis of the enrichment profiles between the MFCP and EHCP groups revealed significant enrichment in the following pathways: *fructose and mannose metabolism* (*p* = 0.003), *arachidonic acid metabolism* (*p* = 0.017), *glycerophospholipid metabolism* (*p* = 0.036), and the *cAMP signaling pathway* (*p* = 0.047).

## 4. Discussion

In animal production, microorganisms and enzymes serve as crucial regulators of material and energy conversion within the organism. Enzymes, acting as biological catalysts, are composed of proteins, amino acids, minerals, and vitamins, participating in the body’s growth and metabolism [37]. Exogenous enzyme treatment can compensate for deficiencies in natural enzymes and enhance nutrient absorption. Microorganisms serve as a bridge between the environment and the host, not only degrading proteins and secreting various enzymes and metabolites [38], but also colonising the gastrointestinal tract to assist young ruminants in establishing beneficial rumen and intestinal microbial communities. The current investigation revealed no notable differences in body weight changes or diarrhea frequency among MFCP-fed and EHCP-fed lambs, demonstrating that these processing approaches have equivalent impacts on suckling lamb development. This could be ascribed to the phenomenon that throughout the fermentation procedure, microorganisms effectively degrade anti-nutritional factors present in cottonseed protein while simultaneously breaking down macromolecular proteins into low molecular weight peptides and amino acids [39], hence augmenting protein breakdown and nutritional uptake in suckling lambs. Alternatively, enzymatic systems demonstrate elevated performance rates and targeted substrate discrimination, enabling them to rapidly and selectively hydrolyze proteins. Throughout the initial digestive stages in young sheep, oligopeptides and liberated amino acids demonstrate superior absorption efficiency compared to native protein molecules, ultimately leading to effects comparable to those observed with fermented cottonseed protein.

Blood biochemical indices function as crucial markers reflecting the metabolic status and nutritional processes in animal species. The circulating levels of TP, ALB, and GLO represent vital parameters indicating the homeostatic balance of protein biosynthesis and breakdown processes in vivo [40]. BUN, the final metabolite of protein catabolism, is closely associated with both the intake and utilization efficiency of dietary protein and reflects the equilibrium between overall protein metabolism and amino acid turnover [41]. Furthermore, serum concentrations of TC, TG, HDL-C, and LDL-C are key markers of lipid metabolic homeostasis, indicating the status of cholesterol and triglyceride synthesis, transportation, and degradation within the organism [42,43]. Serum total bilirubin (TBIL) plays a crucial role in assessing hepatic function, hemolytic status, and biliary patency. Elevated levels of total bilirubin are typically indicative of hepatocellular injury or impaired bile excretion. Hepatic cell damage can further disrupt protein synthesis and ammonia metabolism, ultimately resulting in malnutrition and metabolic imbalance [44]. ALT and AST are key enzymatic indicators used to evaluate hepatic function. The current research revealed no substantial disparities among fermentative and enzymatic hydrolysis groups concerning the above-described blood biochemical markers, implying that both bacterial fermentation and enzymatic breakdown of cottonseed protein yield equivalent influences on the physiological processes in suckling offspring. Glucose is a critical marker for evaluating carbohydrate absorption, transport, and metabolism in ruminants. In the circulation of ruminant animals, glucose is chiefly synthesized via gluconeogenic mechanisms involving branched-chain amino acids arising from rumen bacterial fermentation activities [45]. Within the current investigation, plasma glucose levels in the MFCP group exhibited markedly elevated values compared with those observed in the EHCP group. This difference may be attributable to enzymatic digestion producing peptides that lower blood glucose levels in fasted animals [46].

The rumen microbiota of ruminants has co-evolved with the host, continuously producing VFAs through fermentation, thereby forming a complex regulatory network [47,48]. VFAs arising from microbial fermentative processes within the rumen act both as fundamental energy resources for the host and as regulators of host genetic expression patterns (e.g., *MAPK1*, *PIK3CB*), promoting ruminal epithelial development [49]. In the current experiment, the MFCP treatment group presented considerably elevated ruminal contents of total VFAs, acetate, propionate, butyrate, and valerate in suckling lambs versus the EHCP treatment group. This phenomenon may result from the immediate synthesis of acetate by lactic acid bacteria throughout the fermentative processes [50], while *Saccharomyces cerevisiae*, acting as a probiotic, may modulate the composition and activity of the microbial ecosystem [51], thereby synergistically enhancing propionate production. Propionate, through hepatic gluconeogenesis, forms a positive feedback loop with elevated blood glucose levels. Previous studies have shown that the establishment of the rumen microbiota in young ruminants involves maternal and environmental transmission, followed by rapid microbial interactions within the first six weeks after birth [52]. Early intervention in the rumen microbial community may, therefore, improve health and growth performance in ruminants, exerting long-term beneficial effects [53]. On the other hand, although alkaline protease can efficiently degrade macromolecular proteins, the resulting hydrolysate exhibits a mildly acidic pH of 6.33, which may disrupt the acidic environment of the rumen. This alteration can lead to protein precipitation near their isoelectric points, thereby reducing protein bioavailability and inhibiting the acid-producing activity of rumen microorganisms. Previous studies have demonstrated that maintaining a stable NH_3_-N concentration is critical for minimizing nitrogen loss via the hepatic urea cycle while ensuring efficient microbial protein synthesis [54]. Furthermore, isobutyrate and isovalerate can stimulate the synthesis of branched-chain amino acids and microbial protein within ruminal environments. This study revealed no substantial variations in nitrogen metabolism balance or branched-chain volatile fatty acid synthesis within the rumen of suckling lambs when contrasting MFCP with EHCP treatments. Although no significant differences were observed in growth performance, fermented cottonseed protein may offer specific advantages for ruminal development in lambs compared to the enzymatically hydrolyzed form.

As a specialized foregut organ characteristic of ruminant species, the rumen maintains a diverse microbial population affected by dietary formulation, nutritional parameters, and livestock management factors. Among rumen microorganisms, bacteria account for approximately 95% of the total population [55]. Alpha (*α*) diversity represents a metric employed to assess microbial abundance and taxonomic diversity, whereas beta (*β*) diversity demonstrates variations in microbial community structure across samples [56]. This study found no significant differences in α-diversity metrics between treatment groups. Although the EHCP group exhibited more unique ASVs (727 vs. 569), β-diversity measures were significantly elevated in the MFCP group compared to the EHCP group, indicating greater variation in community structure among samples within the MFCP group. At the taxonomic phylum level, *firmicutes* and *bacteroidota* emerge as the most prevalent bacterial groups in ruminant rumen microbial assemblages [57], executing key roles in cellulose breakdown and polysaccharide metabolism [58,59]. These two phyla also predominated in the EHCP group. In contrast, the MFCP group exhibited *actinobacteriota* as the primary dominant phylum, which was significantly more abundant than in the EHCP. The relative increase in actinomycete abundance contrasts with the conventional rumen microbial community structure, and the core factors underpinning this dominance remain unclear. This finding opens new avenues for future research, which will advance investigations into the driving mechanisms and functional roles of actinomycetes within specific dietary contexts. Detailed genus-level investigation indicated that the MFCP group exhibited markedly higher levels of *pseudoscardovia* and *erysipelotrichaceae_UCG-002*. *erysipelotrichaceae_UCG-002*, a yet-uncategorized genus within the phylum *firmicutes*, and *pseudoscardovia*, a genus within *actinobacteriota* and the family *bifidobacteriaceae*, are important constituents of Gram-positive rumen bacteria [60]. *pseudoscardovia* may degrade oligosaccharides into acetate and lactate via the phosphoketolase pathway [61,62], consistent with the observed increase in acetate concentration in the MFCP group. Taken together, these results suggest that relative to EHCP, MFCP more effectively accelerates rumen development in suckling lambs.

Digestive enzymes are essential components of the animal digestive system, primarily responsible for breaking down macronutrients in the diet into absorbable small molecules, thereby supplying the nutrients necessary for maintenance and growth. Enzyme activity reflects, to some extent, the digestive capacity of the gastrointestinal tract, and a positive correlation exists between enzyme activity and body growth in young animals [63]. Previous studies have shown that feeding SBM hydrolyzed by exogenous enzymes can enhance intestinal digestive enzyme activity [64]. The current study revealed that the enzymatic hydrolysis group of lambs displayed substantially greater *α*-amylase activity in the abomasum and jejunum when contrasted with the fermentation group. This indicates that EHCP using exogenous enzymes is more effective than MFCP in enhancing digestive enzyme activity, consequently facilitating superior nutrient breakdown and uptake in the digestive tract of suckling lambs. The possible mechanism is that enzymatic hydrolysis alters the structural conformation of cottonseed protein, reducing its interaction with gut microbiota, which in turn lowers the formation of resistant starch and indirectly enhances starch digestibility in the gastrointestinal tract. Furthermore, results from this investigation showed that trypsin enzymatic activity was markedly greater in the EHCP group when compared with the MFCP group. Such results might stem from free gossypol contained in cottonseed protein, leading to direct suppression of particular protease enzymes. Through interaction with the unbound *N*-terminal amino groups of lysine within the digestive tract, gossypol may stimulate an increase in trypsin concentration [65,66]. Moreover, the increased *α*-amylase activity in the abomasum facilitates the degradation of lactose in suckling lambs, while the enhanced trypsin activity in the jejunum promotes the conversion of proteins into bioactive oligopeptides. In mammals, dietary proteins and starches are primarily digested by trypsin and *α*-amylase, respectively [67]. Therefore, from the perspective of gastrointestinal digestive enzyme activity, EHCP is more conducive to nutrient absorption in the intestines of suckling lambs than MFCP.

The intestinal tract serves as an essential organ for digestive processes, with intestinal wellness being crucial to the accelerated growth and maturation of lambs. The intestinal mucosal immune system is a crucial component of the body’s overall immunity, capable of secreting both specific and nonspecific substances to form an immune barrier within the gut. Such a defensive mechanism blocks the invasion of disease-causing bacteria while providing protection for sustaining intestinal integrity [68], which holds substantial relevance for the metabolic and defense systems in suckling lambs. The present investigation revealed that both fermented and enzymatically hydrolyzed cottonseed protein produced no notable impacts on sIgA and IgM concentrations within the jejunal mucosa of suckling lambs. Nonetheless, the MFCP treatment group exhibited markedly superior IgG quantities when compared with the EHCP group. This may be attributed to the immunomodulatory effects of bioactive peptides generated during fermentation, which are potentially more potent than those produced through enzymatic hydrolysis. Intestinal immune defense relies not only on antibody-mediated humoral immunity but also on physical barriers and microbial activity within the gut [69]. Among these, tight junctions represent the most critical form of intercellular connection between intestinal mucosal epithelial cells. Constructed from transmembrane and cytoplasmic scaffold proteins, tight junctions fulfill an indispensable function in regulating intestinal mucosal permeability and epithelial barrier integrity [70]. These structures also participate in the specific modulation of small-molecule and ion movement across epithelial tissues [71]. Claudins, Occludin, and the zonula occludens (ZO) family of proteins are key members of the tight junction complex and are essential for maintaining the differential distribution of substances across epithelial cells as well as preserving cell polarity [72,73]. Previous research has established that substituting fermented cottonseed meal for fish meal can boost digestive enzyme performance while concomitantly elevating ZO-1, Occludin, and Claudin-1 expression levels [18]. In this investigation, Occludin and Claudin-1 transcriptional levels within the intestinal mucosa were notably increased in the MFCP group. This could be explained by the probiotic capacity to competitively block pathogenic microbial establishment, thereby indirectly reinforcing intestinal barrier functionality [74]. Increased expression of tight junction proteins signifies enhanced barrier function, aiding in the prevention of harmful substances from traversing the intestinal epithelial barrier. This helps to mitigate damage to the gut and the resulting inflammatory response.

Acting as an essential protein ingredient for animal feed formulations, cottonseed protein shows structural variations through fermentation and enzymatic hydrolysis treatments, which break down large protein molecules to produce short peptides and individual amino acids. This conversion aids in the metabolism of peptides and amino acids and may also reduce the metabolic burden on the liver, thereby reducing liver damage [75,76]. As the central organ of systemic metabolism, the liver plays a pivotal role in nutrient metabolism. Hepatic metabolites, acting as messengers between the host and its internal environment, reflect the organism’s metabolic state in response to nutrients and are critical regulators of energy homeostasis and overall metabolic processes [77]. In this study, differential metabolite profiling and analysis through KEGG pathway enrichment indicated that fermentative microorganisms substantially elevated unsaturated fatty acid compounds such as palmitoleic acid and conjugated linoleic acids (CLA), which are commonly associated with fatty acid storage [78]. In contrast, enzymatic hydrolysis led to a significant downregulation of carbohydrate metabolites such as D-fructose-6-phosphate, suggesting a potential suppression of the glycolytic pathway to reduce energy expenditure [79], thereby promoting growth in suckling lambs.

Additional analysis via KEGG pathway enrichment confirmed that the fermentation-treated group demonstrated considerable activation of arachidonic acid metabolism and the cAMP signaling pathway, implying that microbial fermentation may enhance growth by modulating inflammatory responses and promoting cell proliferation signals [80]. On the other hand, the enzymatic hydrolysis group exhibited significant enrichment in fructose and mannose metabolism as well as glycerophospholipid metabolism, which may support cellular structural stability by regulating lipid synthesis and membrane fluidity [81]. In summary, microbial fermentation of cottonseed protein may promote growth by enhancing anti-inflammatory metabolism and fatty acid biosynthesis, whereas enzymatic hydrolysis supports homeostasis through modulation of carbohydrate metabolism and membrane lipid composition. These differences are likely attributable to microbial metabolites produced during fermentation and structural alterations of cottonseed protein induced by enzymatic hydrolysis.

## 5. Conclusions

This study preliminarily indicates that fermented cottonseed protein, compared to enzymatically hydrolysed cottonseed protein, exhibits enhanced concentrations of ruminal volatile fatty acids (e.g., acetate, propionate), strengthened intestinal barrier function (Occludin and Claudin-1), and modulated fatty acid metabolic pathways. Compared to fermented cottonseed protein, enzymatically hydrolysed cottonseed protein increased α-amylase and trypsin activity in lamb rumen and jejunal contents. However, owing to the short experimental period and limited sample size in lamb trials, no differences were observed between fermented and hydrolysed cottonseed protein in lamb growth performance. Comparatively, the fermented group promoted rumen development and immune regulation in lambs, while the enzymatically hydrolysed group enhanced gastrointestinal digestive enzyme activity. In summary, this study provides theoretical support for the differentiated application of cottonseed protein. Future lamb starter feeds may achieve precise ingredient selection based on production objectives, offering new insights for developing functional protein raw materials. However, this research is limited to comparing two processing methods and does not include a comparison with soybean meal.

## Figures and Tables

**Table 1:** Nutritional components of cottonseed protein after fermentation and enzymatic hydrolysis. (dry matter basis).

Item	MFCP	EHCP
Crude Protein	59.62	60.13
Acid-soluble protein (%)	10.95	21.63
Reduced Sugar (mg/g)	18.11	17.44
pH	4.58	6.33
Free gossypol (mg/kg)	195.30	166.21

MFCP: Microbial Fermentation of Cottonseed Protein; EHCP: Enzymatic Hydrolysate of Cottonseed Protein.

**Table 2:** Composition and nutritional levels of starter feed for suckling lambs (dry matter basis).

Item	MFCP	EHCP
Ingredients (%)		
Corn	58.00	58.00
MFCP	25.00	-
EHCP	-	25.00
Wheat bran	7.00	7.00
Molasses	5.50	5.50
Limestone	0.80	0.80
NaCl	0.70	0.70
Premix ^1^	3.00	3.00
Total	100.00	100.00
Nutrient levels ^2^		
DE (MJ/kg)	16.83	16.87
CP (%)	16.03	15.34
NDF (%)	6.69	6.84
ADF (%)	5.32	5.10
Ca (%)	0.51	0.51
P (%)	0.43	0.43

^1^ Premix per kilogram of daily feed provides: VA, 8000 IU; VD3, 800 IU; VE, 27 IU; copper, 10 mg; iron, 35 mg; zinc, 45 mg; manganese, 45 mg; iodine, 0.4 mg; selenium, 0.4 mg; cobalt, 0.2 mg. ^2^ Nutritional levels are measured values. MFCP: Microbial Fermentation of Cottonseed Protein; EHCP: Enzymatic Hydrolysate of Cottonseed Protein.

**Table 3:** Primer information for qPCR.

Target Gene	Accession ID	Sequence (5′~3′)	Product Length (bp)
ZO-1	XM_060401409.1	F: CGGGAGAGACAAGATGTCCG	163
		R: CCCGCTCTGGAAATGTGGAT	
Claudin-1	NM_001185016.1	F: CATCTTTGTGGCCACCCTTG	99
		R: AGAAAGATCACGCCCCCAAA	
Occludin	XM_060400238.1	F: AGATCAAGTGAGCACCGACC	87
		R: ATGGCAATGCAATTCATCAGG	
*β*-actin	XM_060405599.1	F: TGCTTCCTTCTCTCTCTCCAGAT	123
		R: GTACTCCTGCTTGCTGATCCA	

**Table 4:** Effects of fermentation and enzymatic hydrolysis of cottonseed protein on body weight changes in suckling lambs.

Items	MFCP	EHCP	SEM	*p*-Value
Initial weight (kg)	5.15	5.38	0.211	0.612
Final weight (kg)	11.08	10.78	0.362	0.701
Average daily gain (g/d)	113.93	103.81	6.814	0.484
Diarrhea rate (%)	1.28	1.28	0.641	1.000

SEM: standard error of the mean; *p* < 0.01 indicates a highly significant difference, *p* < 0.05 indicates a significant difference, and 0.05 ≤ *p* < 0.10 indicates a significant trend toward difference. MFCP: Microbial Fermentation of Cottonseed Protein; EHCP: Enzymatic Hydrolysate of Cottonseed Protein.

**Table 5:** Effects of fermentation and enzymatic hydrolysis of cottonseed protein on serum biochemical parameters in suckling lambs.

Items	MFCP	EHCP	SEM	*p*-Value
TP (g/L)	57.12	59.47	2.578	0.670
ALB (g/L)	29.38	26.87	1.107	0.291
GLB (g/L)	27.73	32.60	2.806	0.412
BUN (mmol/L)	7.68	8.37	0.309	0.282
Total Bilirubin (µmol/L)	2.30	3.38	0.486	0.296
GLU (mmol/L)	4.38	1.46	0.534	<0.001
ALT (U/L)	13.00	11.17	1.026	0.202
AST (U/L)	81.33	77.50	5.025	0.361
T-CHO (mmol/L)	1.12	1.09	0.038	0.155
TG (mmol/L)	0.27	0.24	0.056	0.652
HDL-c (mmol/L)	0.69	0.62	0.037	0.336
LDL-c (mmol/L)	0.38	0.34	0.022	0.435

SEM: standard error of the mean; *p* < 0.01 indicates a highly significant difference, *p* < 0.05 indicates a significant difference, and 0.05 ≤ *p* < 0.10 indicates a significant trend toward difference. TP: total protein; ALB: albumin; GLB: globulin; BUN: urea nitrogen; GLU: glucose; ALT: alanine aminotransferase; AST: aspartate aminotransferase; T-CHO: total cholesterol; TG: triglycerides; HDL-c: High-density lipoprotein cholesterol; LDL-c: Low-density lipoprotein cholesterol. MFCP: Microbial Fermentation of Cottonseed Protein; EHCP: Enzymatic Hydrolysate of Cottonseed Protein.

**Table 6:** Effects of fermentation and enzymatic hydrolysis of cottonseed protein on rumen fermentation parameters in suckling lambs.

Items	MFCP	EHCP	SEM	*p*-Value
Acetic acid (mmol/L)	37.55	15.40	4.306	0.002
Propionic acid (mmol/L)	9.48	5.24	0.803	0.002
Isobutyric acid (mmol/L)	0.64	0.49	0.044	0.082
Butyric acid (mmol/L)	4.88	2.08	0.501	<0.001
Isovaleric acid (mmol/L)	0.85	0.65	0.068	0.142
Valeric acid (mmol/L)	2.02	0.75	0.228	0.003
A/P	3.93	2.94	0.296	0.064
Total VFAs (mmol/L)	55.42	24.61	5.739	0.001
NH_3_-N (mmol/L)	0.69	0.66	0.022	0.405
Lactic acid (mmol/L)	0.55	0.59	0.033	0.552

SEM: standard error of the mean; *p* < 0.01 indicates a highly significant difference, *p* < 0.05 indicates a significant difference, and 0.05 ≤ *p* < 0.10 indicates a significant trend toward difference. A/P: Acetic acid/Propionic acid; Total VFAs: Total volatile fatty acids. MFCP: Microbial Fermentation of Cottonseed Protein; EHCP: Enzymatic Hydrolysate of Cottonseed Protein.

**Table 7:** Effects of fermentation and enzymatic hydrolysis of cottonseed protein on gastrointestinal digestive enzyme activities in suckling lambs.

Items	MFCP	EHCP	SEM	*p*-Value
Abomasum				
α-Amylase (U/g)	163.98	266.04	26.397	0.047
Lipase (U/g)	275.59	349.43	69.341	0.617
Trypsin (U/mg)	104.98	105.48	4.334	0.957
Duodenum				
α-Amylase (U/g)	262.84	465.85	59.766	0.117
Lipase (U/g)	114.40	249.76	40.740	0.116
Trypsin (U/mg)	778.79	575.07	105.447	0.358
Jejunum				
α-Amylase (U/g)	243.00	371.27	28.752	0.011
Lipase (U/g)	81.72	171.26	24.131	0.059
Trypsin (U/mg)	298.39	786.42	105.212	0.013

SEM: standard error of the mean; *p* < 0.01 indicates a highly significant difference, *p* < 0.05 indicates a significant difference, and 0.05 ≤ *p* < 0.10 indicates a significant trend toward difference. MFCP: Microbial Fermentation of Cottonseed Protein; EHCP: Enzymatic Hydrolysate of Cottonseed Protein.

## Data Availability

The raw data supporting the conclusions of this article will be made available by the authors upon request.

## Supplementary Materials

The following supporting information can be downloaded at: https://www.mdpi.com/article/10.3390/ani15182652/s1, Table S1. Amino acid content of cottonseed protein after fermentation and enzymatic hydrolysis. Table S2. Effects of Fermented and Enzymatically Hydrolysate Cottonseed Protein on the Relative Abundance of the Top 10 Microbial Taxa at the Phylum Level in the Rumen of Suckling Lambs. Table S3. Effects of Fermented and Enzymatically Hydrolysate Cottonseed Protein on the Relative Abundance of the Top 10 Microbial Taxa at the Genus Level in the Rumen of Suckling Lambs. Table S4. Effects of Fermented and Enzymatically Hydrolyzed Cottonseed Protein on the Expression of Immunoglobulins and Tight Junction Protein mRNA in Jejunal Mucosa of Suckling Lambs. Table S5. Differentially Upregulated Compounds in Liver Metabolism of Suckling Lambs Fed Fermented and Enzymatically Hydrolyzed Cottonseed Protein. Table S6. Differentially Downregulated Compounds in Liver Metabolism of Suckling Lambs Fed Fermented and Enzymatically Hydrolyzed Cottonseed Protein.

## Abbreviations

The following abbreviations are used in this manuscript:

MFCP	Microbial Fermentation of Cottonseed Protein
EHCP	Enzymatic Hydrolysate of Cottonseed Protein
SBM	Soybean meal
CSM	Cottonseed meal
TP	Total protein
ALB	Albumin
GLB	Globulin
BUN	Urea nitrogen
GLU	Glucose
ALT	Alanine aminotransferase
AST	Aspartate aminotransferase
T-CHO	Total cholesterol
TG	Triglycerides
HDL-c	High-density lipoprotein cholesterol
LDL-c	Low-density lipoprotein cholesterol
VFAs	Volatile fatty acids
sIgA	Secretory Immunoglobulin A
IgG	Immunoglobulin G
IgM	Immunoglobulin M
FC	Fold change
CLA	Conjugated linoleic acids
19S-HETE	19S-Hydroxyeicosatetraenoic acid
13(S)-HODE	13(S)-Hydroxyoctadecadienoic acid
