# Supplementary material for: Effects of Fermentation and Enzymatic Hydrolysis of Cottonseed Protein on Rumen Fermentation Characteristics, Intestinal Barrier Function, and Hepatic Metabolism in Suckling Lambs
Source: Animals (Basel). 2025 Sep 10;15(18):2652. doi: 10.3390/ani15182652 (PMC12466640; doi:10.3390/ani15182652)
Supplement: Supplementary file 1 [file animals-15-02652-s001.zip › animals-3851469-supplementary.pdf]

Supplemental materials

Effects of Fermentation and Enzymatic Hydrolysis of Cottonseed Protein on Growth Performance, Barrier Function, and Hepatic Metabolism in Suckling Lambs

Table S1. Amino acid content of Cottonseed Protein after Fermentation and Enzymatic Hydrolysis

Item	MFCP	EHCP
Amino acid content (%)		
Aspartic acid	5.56	4.97
Threonine	1.89	1.76
Serine	2.55	2.39
Glutamic acid	12.82	12.17
Proline	2.1	2.04
Glycine	2.4	2.23
Alanine	2.25	2.13
Cystine	0.45	0.63
Valine	2.44	2.33
Methionine	0.57	0.52
Isoleucine	1.72	1.65
Leucine	3.30	3.10
Tyrosine	1.63	1.63
Phenylalanine	3.13	3.00
Histidine	1.70	1.59
Lysine	2.45	2.29
Arginine	7.49	6.28
Total amino acids	54.46	50.71

MFCP: Microbial Fermentation of Cottonseed Protein; EHCP: Enzymatic Hydrolysate of Cottonseed Protein.

Table S2. Effects of Fermented and Enzymatically Hydrolysate Cottonseed Protein on the Relative Abundance of the Top 10 Microbial Taxa at the Phylum Level in the Rumen of Suckling Lambs

Items	MFCP	EHCP	SEM	p-value
Actinobacteriota	0.3531	0.1082	0.060	0.041
Firmicutes	0.3022	0.3321	0.051	0.699
Bacteroidota	0.2677	0.3713	0.051	0.589
Proteobacteria	0.0113	0.1458	0.038	0.132
Euryarchaeota	0.0437	0.0177	0.009	0.180
Patescibacteria	0.0170	0.0197	0.009	0.937
Spirochaetota	0.0004	0.0011	0.003	0.041
Fibrobacterota	0.0000	0.0018	0.001	0.394
Desulfobacterota	0.0020	0.0012	0.001	0.310
Cyanobacteria	0.0025	0.0006	0.001	0.240

SEM: standard error of the mean; $p < 0.01$ indicates a highly significant difference, $p < 0.05$ indicates a significant difference, and $0.05 \leqslant p < 0.10$ indicates a significant trend toward difference. MFCP: Microbial Fermentation of Cottonseed Protein; EHCP: Enzymatic Hydrolysate of Cottonseed Protein.

Table S3. Effects of Fermented and Enzymatically Hydrolysate Cottonseed Protein on the Relative Abundance of the Top 10 Microbial Taxa at the Genus Level in the Rumen of Suckling Lambs

Items	MFCP	EHCP	SEM	p-value
Lachnospiraceae_NK3A20_group	0.0197	0.1259	0.051	0.589
Prevotella_7	0.2320	0.2046	0.047	1.000
Olsenella	0.1002	0.0140	0.034	0.132
Pseudoscardovia	0.2485	0.0928	0.035	0.041
Succinivibrio	0.0108	0.1432	0.038	0.093
Erysipelotrichaceae_UCG-002	0.1122	0.0172	0.024	0.026
Dialister	0.0563	0.0230	0.013	0.394
Megasphaera	0.0103	0.0361	0.013	0.240
Succiniclasticum	0.0004	0.0237	0.012	0.132
Prevotella	0.0004	0.0272*	0.010	0.041

SEM: standard error of the mean; $p < 0.01$ indicates a highly significant difference, $p < 0.05$ indicates a significant difference, and $0.05 \leqslant p < 0.10$ indicates a significant trend toward difference. MFCP: Microbial Fermentation of Cottonseed Protein; EHCP: Enzymatic Hydrolysate of Cottonseed Protein.

Table S4. Effects of Fermented and Enzymatically Hydrolyzed Cottonseed Protein on the Expression of Immunoglobulins and Tight Junction Protein mRNA in Jejunal Mucosa of Suckling Lambs

Items	MFCP	EHCP	SEM	p-value
SIgA (μg/mL)	1.60	1.66	0.019	0.154
IgG (mg/mL)	17.58	15.07	0.640	0.043
IgM (mg/mL)	0.060	0.064	0.015	0.111
ZO-1	3.66	3.98	0.228	0.511
Claudin-1	2.14	1.42	0.153	0.010
Occludin	0.98	0.69	0.058	0.009

SEM: standard error of the mean; $p < 0.01$ indicates a highly significant difference, $p < 0.05$ indicates a significant difference, and $0.05 \leqslant p < 0.10$ indicates a significant trend toward difference. SIgA: Secretory Immunoglobulin A; IgG: Immunoglobulin G; IgM: Immunoglobulin M. MFCP: Microbial Fermentation of Cottonseed Protein; EHCP: Enzymatic Hydrolysate of Cottonseed Protein.

Table S5. Differentially Upregulated Compounds in Liver Metabolism of Suckling Lambs Fed Fermented and Enzymatically Hydrolyzed Cottonseed Protein

Compound ID	Compound Name	Formula	Trend	Fold change	Pvalue
Com_937_neg	5-Aminonicotinic acid	C6H6N2O2	up	2.376	0.000
Com_544_neg	trans-10-Nonadecenoic acid (C19-1T)	C19H36O2	up	2.902	0.000
Com_1917_neg	2-keto palmitic acid	C16H30O3	up	4.296	0.000
Com_2350_neg	3-(8,11,14-Pentadecatrieny l) phenol	C21H30O	up	2.483	0.000
Com_643_neg	Conjugated linoleic acids (CLA)	C18H32O2	up	2.982	0.000
Com_1564_neg	Aplotaxene	C17H28	up	1.989	0.000
Com_414_neg	Methyl pentadecanoate	C16H32O2	up	4.077	0.000
Com_3423_neg	FAHFA 36:1	C36H68O4	up	4.563	0.000
Com_3419_neg	FAHFA 36:3	C36H64O4	up	4.582	0.001
Com_611_neg	Glaucocalyxin A	C20H28O4	up	2.510	0.001
Com_1681_neg	O-pivaloylcarnitine	C12H23NO4	up	1.940	0.001
Com_597_neg	Bergenin	C14H16O9	up	1.653	0.001
Com_2806_neg	N-Stearoyl Alanine	C21H41NO3	up	8.256	0.001
Com_2216_neg	12-(2,3-Dihydroxycyclopentyl)-2-dodecanone	C17H32O3	up	2.210	0.001
Com_2325_neg	(1S,2R)-3-oxo-2-pentylcyclopentane-1-octanoic acid	C18H32O3	up	1.824	0.001
Com_2557_neg	19S-HETE	C20H32O3	up	2.733	0.001
Com_3752_pos	Penazetidine A	C23H47NO2	up	8.640	0.001
Com_2829_neg	N-Eicosapentaenoyl Glycine	C22H33NO3	up	3.860	0.001
Com_2628_pos	3-Oxotetradecanoic acid	C14H26O3	up	3.114	0.001
Com_2771_neg	Carbocyclic thromboxane A2	C22H36O3	up	2.358	0.002
Com_610_neg	Palmitoylglycine	C18H35NO3	up	4.918	0.002
Com_1769_neg	Meteloidine	C13H21NO4	up	2.744	0.002
Com_2778_neg	11-deoxy-11-methylene-PGD2	C21H34O4	up	1.709	0.002
Com_3132_neg	N-Arachidonoyl Histidine	C26H39N3O3	up	8.015	0.002
Com_2670_neg	(+)-Rauflexine	C21H24N2O2	up	6.919	0.002
Com_1343_pos	Arachidonoyl ethanolamide	C22H37NO2	up	4.419	0.002

Com_3279_pos	4-Hydroxy-estazolam	C16H11ClN4O	up	5.216	0.002
Com_2527_neg	7-Oxo-8,15-isopimaradien-18-oic acid	C20H28O3	up	1.705	0.002
Com_2782_neg	Melophlin E	C21H37NO3	up	3.466	0.002
Com_3056_neg	N-Oleoyl Histidine	C24H41N3O3	up	8.600	0.002
Com_2996_neg	N-Arachidonoyl Valine	C25H41NO3	up	9.283	0.002
Com_2382_pos	(+)-trans-alpha-Irone	C14H22O	up	1.513	0.002
Com_2873_neg	N-Palmitoyl Asparagine	C20H38N2O4	up	5.651	0.002
Com_407_neg	Palmitoleic acid	C16H30O2	up	2.919	0.002
Com_1644_neg	6-Ketomyristic acid	C14H26O3	up	2.028	0.002
Com_1515_neg	5-phospho-beta-D-ribosylaminium (1-)	C5H12NO7P	up	2.931	0.003
Com_1810_neg	2-Methoxytetradecanoic acid	C15H30O3	up	1.695	0.003
Com_634_neg	5-Pentadecylresorcinol	C21H36O2	up	2.247	0.003
Com_3161_neg	N-Arachidonoyl Phenylalanine	C29H41NO3	up	9.040	0.003
Com_2919_neg	N-Palmitoyl Glutamine	C21H40N2O4	up	5.396	0.003
Com_975_neg	3-Trimethylsilylpropionic acid	C6H14O2Si	up	1.811	0.003
Com_2488_neg	FA 18:2+2O	C18H32O4	up	5.934	0.004
Com_3021_neg	N-Linoleoyl Glutamine	C23H40N2O4	up	2.860	0.004
Com_2328_neg	13(S)-HODE	C18H32O3	up	1.579	0.004
Com_2259_neg	ent-16-Kauren-19-ol	C20H32O	up	2.572	0.004
Com_2955_neg	N-Arachidonoyl Serine	C23H37NO4	up	2.712	0.004
Com_3031_neg	(9Z,12Z)-3-hydroxyhexadecadienoylcarnitine	C23H41NO5	up	3.659	0.005
Com_3030_neg	N-Linoleoyl Methionine	C23H41NO3S	up	8.596	0.005
Com_507_neg	Elaidic acid	C18H34O2	up	2.693	0.005
Com_2511_neg	Octadecanedioic acid	C18H34O4	up	5.944	0.005
Com_3270_pos	Methyl (10E,12Z,15Z)-9-hydroxyoctadeca-10,12,15-trienoate	C19H32O3	up	3.605	0.005
Com_2454_neg	Isolinderanolide B	C19H32O3	up	2.001	0.005
Com_3202_neg	N-Eicosapentaenoyl Tyrosine	C29H39NO4	up	3.402	0.005
Com_1134_neg	Hexanethioic acid S-propyl ester	C9H18OS	up	1.652	0.006
Com_2954_pos	Stearidonic Acid	C18H28O2	up	3.302	0.006

Com_3104_neg	N-Arachidonoyl Glutamic acid	C25H39NO5	up	2.634	0.006
Com_2834_pos	(Z)-3-Hexenyl beta-D-glucopyranoside	C12H22O6	up	2.439	0.006
Com_3725_pos	Terminaline	C23H41NO2	up	4.165	0.006
Com_2974_neg	3beta-Hydroxypregn-5-en-20-one sulfate	C21H32O5S	up	2.252	0.006
Com_2868_neg	Semiplenamide F	C22H43NO3	up	12.034	0.007
Com_277_neg	3-hydroxylaurate	C12H24O3	up	2.291	0.007
Com_4149_pos	(10Z)-Nonadec-10-enoylcarnitine	C26H49NO4	up	6.822	0.007
Com_3098_neg	Solanocardinol	C27H45NO3	up	9.114	0.007
Com_1217_pos	Ginkgolic Acid	C22H34O3	up	2.325	0.008
Com_138_neg	1H-Indole-3-acetamide	C10H10N2O	up	1.821	0.008
Com_2528_neg	5-(8,11-Pentadecadienyl)-1,3-benzenediol	C21H32O2	up	2.213	0.008
Com_2732_neg	Oxyphencyclimine	C20H28N2O3	up	4.760	0.009
Com_1619_neg	Chokol F	C14H24O3	up	2.920	0.009
Com_4036_pos	10-MethylHexadecanoylcarnitine	C24H47NO4	up	3.016	0.010

Table S6. Differentially Downregulated Compounds in Liver Metabolism of Suckling Lambs Fed Fermented and Enzymatically Hydrolyzed Cottonseed Protein

Compound ID	Compound Name	Formula	Trend	Fold change	Pvalue
Com_406_neg	Deoxyarbutin	C11H14O3	down	0.582	0.000
Com_2701_neg	A-O-Methyldihydrobotrydialone	C18H28O6	down	0.574	0.000
Com_3000_neg	Glu-Ala-Trp	C19H24N4O6	down	0.289	0.000
Com_531_neg	Narciclasine	C14H13NO7	down	0.180	0.000
Com_1545_pos	LysoPC(14:0/0:0)	C22H46NO7P	down	0.307	0.000
Com_343_neg	N-Lactoyl-Phenylalanine	C12H15NO4	down	0.340	0.000
Com_2018_neg	Hymenoside W	C11H20O8	down	0.153	0.000
Com_3065_neg	Calealactone B	C21H26O9	down	0.355	0.000
Com_2833_pos	L-phenylalanyl-L-proline	C14H18N2O3	down	0.327	0.000
Com_2913_pos	Alibendol	C13H17NO4	down	0.094	0.000
Com_3128_neg	Taraxafolide	C21H28O10	down	0.284	0.000

Com_255_pos	Glycylproline	C7H12N2O3	down	0.442	0.000
Com_2808_neg	GlcAbeta1-6Galbeta	C12H20O12	down	0.176	0.000
Com_3164_neg	Glycerophospho-N-Palmitoyl Ethanolamine	C21H44NO7P	down	0.330	0.000
Com_2880_pos	H-Ser-Tyr-OH	C12H16N2O5	down	0.368	0.000
Com_2969_pos	Prolyl-tyrosine	C14H18N2O4	down	0.404	0.000
Com_3240_pos	Leu-Thr-Ala	C13H25N3O5	down	0.177	0.000
Com_439_neg	Indirubin	C16H10N2O2	down	0.266	0.001
Com_2635_pos	Glutaminylproline	C10H17N3O4	down	0.192	0.001
Com_1668_neg	Ile Leu	C12H24N2O3	down	0.375	0.001
Com_3135_neg	Paeonenoide C	C28H42O4	down	0.275	0.001
Com_3059_pos	Arg Leu	C12H25N5O3	down	0.416	0.001
Com_2740_neg	Leu Ile Thr	C16H31N3O5	down	0.348	0.001
Com_831_pos	DL-Leucyl-DL-phenylalanine	C15H22N2O3	down	0.293	0.001
Com_3250_pos	Thr-Trp	C15H19N3O4	down	0.187	0.001
Com_2306_neg	Tyr Leu	C15H22N2O4	down	0.323	0.001
Com_1750_neg	1-Carboxyethyltyrosine	C12H15NO5	down	0.379	0.001
Com_1344_neg	Valylserine	C8H16N2O4	down	0.525	0.001
Com_3540_pos	gamma-Glutamyl-S-methylcysteinyl-beta-alanine	C12H21N3O6S	down	0.286	0.001
Com_3881_pos	Arg Ile Val	C17H34N6O4	down	0.257	0.001
Com_3013_pos	Thr-Tyr	C13H18N2O5	down	0.306	0.001
Com_677_pos	Phenylalanylvaline	C14H20N2O3	down	0.370	0.001
Com_1890_neg	Portulacaxanthin III	C11H12N2O6	down	0.440	0.001
Com_3292_pos	H-PHE-PHE-OH	C18H20N2O3	down	0.301	0.001
Com_2978_pos	Phe Asn	C13H17N3O4	down	0.279	0.001
Com_2739_neg	Asp Val Leu	C15H27N3O6	down	0.420	0.001
Com_324_neg	L-Leucyl-L-Valine	C11H22N2O3	down	0.445	0.001
Com_3011_neg	Glu-Ile-Phe	C20H29N3O6	down	0.340	0.001
Com_3076_neg	LysoPE(0:0/14:0)	C19H40NO7P	down	0.424	0.001
Com_3493_pos	Val Val Leu	C16H31N3O4	down	0.245	0.001

Com_3695_pos	Leu-Leu-Asn	C16H30N4O5	down	0.539	0.001
Com_2665_pos	Methionyl-Proline	C10H18N2O3S	down	0.108	0.001
Com_3609_pos	Val Ile Leu	C17H33N3O4	down	0.374	0.001
Com_1451_neg	N-lactoyl-Methionine	C8H15NO4S	down	0.445	0.001
Com_3898_pos	(+)-Desoxyscalarin	C25H40O3	down	0.510	0.002
Com_1629_neg	D-myo-Inositol 1,2-cyclic phosphate	C6H11O8P	down	0.414	0.002
Com_2676_pos	Asparaginylaspartic acid	C8H13N3O6	down	0.333	0.002
Com_3012_pos	Tyrosyl-Threonine	C13H18N2O5	down	0.400	0.002
Com_3101_pos	Phe-Ala-Gly	C14H19N3O4	down	0.238	0.002
Com_1876_neg	N3,5'-Cycloxanthosine	C10H10N4O5	down	0.382	0.002
Com_2554_pos	Thr Leu	C10H20N2O4	down	0.425	0.002
Com_862_pos	D-Fructose-6-phosphate	C6H13O9P	down	0.388	0.002
Com_4127_pos	Cryogenine	C26H29NO5	down	0.210	0.002
Com_2908_neg	1-Myristoyl-2-Hydroxy-sn-Glycero-3-Phosphate	C17H35O7P	down	0.213	0.002
Com_2654_neg	sn-Glycero-3-phospho-1-inositol	C9H19O11P	down	0.594	0.002
Com_2656_pos	H-Leu-Asn-OH	C10H19N3O4	down	0.377	0.002
Com_2457_pos	N-Acetyl-D-mannosaminolactone	C8H13NO6	down	0.303	0.002
Com_1236_neg	NALPHA-ACETYL-L-LYSINE	C8H16N2O3	down	0.428	0.003
Com_2897_neg	N-Methyl-2,3,7,8-tetramethoxybenzophenanthridine-6(5H)-one	C22H21NO5	down	0.318	0.003
Com_2269_neg	Altenusin	C15H14O6	down	0.232	0.003
Com_400_pos	D-Fructose	C6H12O6	down	0.206	0.003
Com_2726_neg	Tyr-tyr	C18H20N2O5	down	0.237	0.003
Com_3032_neg	Pterodontoside B	C21H32O8	down	0.404	0.003
Com_2118_pos	D-Alanyl-D-serine	C6H12N2O4	down	0.467	0.003
Com_2797_pos	Lys Leu	C12H25N3O3	down	0.409	0.003
Com_3651_pos	Phe-Ala-Asp	C16H21N3O6	down	0.495	0.003
Com_1283_neg	Desoxy	C11H17NO2	down	0.290	0.003
Com_1830_neg	Asparaginylglutamine	C9H16N4O5	down	0.348	0.003
Com_3727_pos	Isomahubanolide 23	C23H40O3	down	0.378	0.003

Com_1994_pos	4-Acetylimidazo[4,5-c]pyridine	C8H7N3O	down	0.469	0.003
Com_2286_neg	Itoside L	C12H20O8	down	0.117	0.004
Com_3384_neg	LysoPE(0:0/22:1(13Z))	C27H54NO7P	down	0.190	0.004
Com_583_pos	O-Succinyhomoserine	C8H13NO6	down	0.260	0.004
Com_2800_pos	D-Myoinositol 4-phosphate	C6H13O9P	down	0.235	0.004
Com_2855_neg	Ser Ile Phe	C18H27N3O5	down	0.398	0.004
Com_3390_pos	Mequitazine	C20H22N2S	down	0.353	0.004
Com_1924_neg	Carbutamide	C11H17N3O3S	down	0.148	0.004
Com_3321_neg	LysoPE(0:0/20:1(11Z))	C25H50NO7P	down	0.652	0.004
Com_2211_pos	Alanylproline	C8H14N2O3	down	0.155	0.004
Com_4374_pos	Roehybridine	C31H39N3O5	down	0.621	0.005
Com_2828_neg	Ile Ile Asp	C16H29N3O6	down	0.316	0.005
Com_308_neg	3,4-Dihydroxymandelaldehyde	C8H8O4	down	0.398	0.005
Com_2967_neg	Glu-Val-Phe	C19H27N3O6	down	0.459	0.005
Com_4421_pos	LysoPC(22:4(7Z,10Z,13Z,16Z)/0:0)	C30H54NO7P	down	0.390	0.006
Com_3058_neg	7-O-Methylmorroniside	C18H28O11	down	0.161	0.006
Com_984_neg	Ethyl 3-mercaptobutyrate	C6H12O2S	down	0.554	0.006
Com_1520_neg	Prolyl-Asparagine	C9H15N3O4	down	0.191	0.006
Com_2008_pos	Ethyl 3-(methylthio)butanoate	C7H14O2S	down	0.626	0.006
Com_781_neg	Darutoside	C26H44O8	down	0.183	0.006
Com_1522_pos	Mycophenolate Mofetil	C23H31NO7	down	0.158	0.006
Com_2481_pos	Phe Gly	C11H14N2O3	down	0.625	0.006
Com_4287_pos	LysoPC(15:0/0:0)	C23H48NO7P	down	0.533	0.006
Com_2343_neg	5'-S-Methyl-5'-thioinosine	C11H14N4O4S	down	0.389	0.006
Com_2475_pos	Dihydrozeatin	C10H15N5O	down	0.196	0.006
Com_2919_pos	(2R,3R)-3-Methylglutamyl-5-semialdehyde-N6-lysine	C12H23N3O4	down	0.397	0.006
Com_1727_pos	Ammelide	C3H4N4O2	down	0.466	0.007
Com_3833_pos	Angoline	C22H21NO5	down	0.239	0.007
Com_1213_neg	Erinapyrone C	C8H10O5	down	0.302	0.007

Com_1891_neg	2-O-(alpha-D-Mannosyl)-D-glycerate	C9H16O9	down	0.416	0.008
Com_4332_pos	LysoPE(20:4(8Z,11Z,14Z,17Z)/0:0)	C25H44NO7P	down	0.386	0.008
Com_1951_neg	Dikegulac	C12H18O7	down	0.254	0.009
Com_1679_neg	S-3-oxodecanoyl cysteamine	C12H23NO2S	down	0.571	0.009
Com_2983_neg	Hypochoeroside K	C21H34O7	down	0.383	0.009
